# Action steps using ACEs and trauma-informed care: a resilience model

**DOI:** 10.1186/s40352-017-0050-5

**Published:** 2017-04-28

**Authors:** Laurie Leitch

**Affiliations:** Threshold GlobalWorks, New York, NY USA

**Keywords:** Neuroscience, Neuroplasticity, ACE study, Trauma Informed Care (TIC), Resilience, Self-regulation skills

## Abstract

This paper 1) discusses two important contributions that are shaping work with vulnerable and under-resourced populations: Kaiser Permanente’s (1998) Adverse Childhood Experiences Study (ACE) which includes the impact of adverse experiences in childhood on adult health and health behaviors and the more recent advent of what has come to be known as Trauma-Informed Care (TIC), programs which incorporate knowledge of the impact of early trauma into policies and programs. 2) Despite many positive benefits that have come from both contributions there are unintended consequences, described in the paper, that have an impact on research and program evaluation as well as social policies and programs. 3) Three key neuroscience concepts are recommended for inclusion in Trauma-Informed Care programs and practices in ways that can enrich program design and guide the development of practical, resilience-oriented interventions that can be evaluated for outcomes. 4) Finally, a resilience-oriented approach to TIC is recommended that moves from trauma *information* to neuroscience-based *action* with practical skills to build greater capacity for self-regulation and self-care in both service providers and clients. Examples from criminal justice are used.

## Background

The work of clinicians and other service providers who design and implement programs for vulnerable populations has been greatly enhanced by the incorporation of two building blocks of understanding: Kaiser Permanente’s (1998) Adverse Childhood Experiences study (ACE) and the growing use and refinement of a values-based orientation to individuals that draws upon ACE findings called Trauma-Informed Care (TIC).

The ACE study and TIC have generated important strides in helping service providers as well as clients better understand the impact of distressing and traumatic events on a wide range of health indicators and behaviors. Incorporating findings from the ACE study into TIC can reduce the pathologizing of symptomatic behavior by viewing symptoms as normal reactions to abnormal experiences (Evans and Coccoma 2014; Van der Kolk 2014), foster screenings for trauma history during intakes (Harris and Fallot 2001), shape staff practices that strengthen relationships between providers and clients, enhance personal safety, create a sense of welcome and respect in service delivery spaces (Elliott et al. 2005; Harris and Fallot 2001) and inspire delivery of preventive services to vulnerable individuals and families as early as possible.

There are, however, unintended consequences that can be seen in the ways that the ACE study and Trauma-Informed Care have shaped research and service delivery. This article presents a brief overview of these two important contributions and discusses the unintended consequences that can influence practices and programs to the detriment of the very individuals they intend to serve. Recommendations are discussed that include: incorporating key neuroscience concepts into TIC, the use of neuroscience-based self-regulation skills for staff and clients, and a specific framework for designing information gathering processes including research and evaluation as well as client intakes. The framework includes attention to protective experiences and characteristics and promotes research and evaluation design in a way that explicitly is intended to create a rhythm or pattern of questioning that enhances resilience and decreases distress and potential re-traumatization.

## Methods

The paper presents a rationale for expanding TIC to include key neuroscience concepts that can contribute to intake and evaluation processes and skills-based interventions. The intent is translational science that describes the movement of science information into social services and then expands that science from enriched information to concrete skills-based action.

## The Adverse Childhood Experiences (ACE) study

From 1995 to 1997 Kaiser Permanente’s Health Appraisal Clinic, in collaboration with Centers for Disease Control and Prevention, implemented one of the largest studies ever conducted on the origins of risk factors that have negative health and social consequences and the cumulative incidence and influence of psychological and physical abuse including: neglect, sexual abuse, witnessing violence, exposure to substance abuse, mental illness, suicidal behavior, and imprisonment of a family member (independent variables) on dependent variables that were measures of both mental health (depression, suicidality) and physical health (heart disease, cancer, chronic lung disease, skeletal fractures, liver disease, obesity) and health-related behaviors (alcoholism, drug abuse, smoking, high numbers of sexual partners) and poor self-rated health (Felitti et al. 1998).

The ACE questionnaire was constructed using selected questions from published surveys (American Journal of Preventive Medicine 2017). Prior to the survey there had been little study of the relationship between early childhood adverse experiences and adult medical problems and behaviors (Felitti et al. 1998).

The ACE survey data was collected by mail from two waves of a sample of 17,000 adult members of Kaiser’s Health Maintenance Organization in San Diego, California between 1995 and 1997. The sample size itself was impressive. The release of the study findings was shocking to many when they showed the extent to which adverse childhood events negatively shaped future social and physical health outcomes, including life expectancy.

Perhaps less surprising, the findings showed that the more negative events a child experienced the higher the likelihood s/he had as an adult of suffering an array of health and behavior problems including alcoholism, chronic pulmonary disease, depression, illicit drug use, liver disease, adolescent pregnancy and many more (Centers for Disease Control and Prevention 2014a, b). Further, adults with the highest level of ACEs had a life expectancy 20 years less than those without high levels of ACEs. The study sample did not consist primarily of low-income minority adults, a demographic often found to be “at risk.” It was mainly comprised of white, middle and upper income employed people; people who might be expected to have had more stable childhood environments because of parents’ employment and income.

The original ACE study has generated more than 70 scientific articles, scores of conference presentations, and has shaped the design of research and as well as social programs. It is beyond the scope of this article to present a comprehensive review of the studies of the ACE survey, but ACEs Too High (2017) provides a list of ACE studies by year.

Studies using the ACE questionnaire have expanded beyond Kaiser’s sample of white, HMO patients to include, for example, special populations such as children of alcoholics (Dube et al. 2001), and children with an incarcerated parent (Geller et al. 2009) and have found higher prevalences of ACEs than in the original Kaiser sample.

ACE Studies of justice-involved populations (Baglivio et al. 2014; Messina and Grella 2006; Miller and Najavits 2012; Reavis et al. 2013) including juvenile justice-involved youth (Dierkhising et al. 2013) are raising awareness of the association of early childhood trauma and offender behaviors and needs, as are studies of justice-involved samples that include a focus on childhood trauma without using the ACE questionnaire (Wolff and Shi 2012). The studies consistently find elevated rates of childhood trauma in incarcerated populations and offender groups. For example, the Reavis et al. study (2013) of incarcerated males found ACE scores above 4 to be four times higher than in a normative male population.

By bringing attention to the powerful impact that negative childhood experiences have on future health and functioning, the ACE study demonstrates the importance of gathering information early in the lives of children and their families and designing early intervention programs that target violence and neglect. It also points to the importance of collecting trauma histories from clients and highlights the essential role of prevention in program design. A particularly important contribution the Ace survey has made to offender and incarcerated groups is to emphasize the importance of trauma-targeted interventions in jails and prisons as well as in diversion programs.

The ACE study has inspired other large-scale, risk-oriented CDC-sponsored health surveys such as The Family Health History and Health Appraisal Questionnaires and The Behavioral Risk Factor Surveillance System (BRFSS) that focus on childhood maltreatment and household dysfunction. The BRFSS is now conducted by telephone in all 50 states, the District of Columbia and 3 U.S. territories, making it the “largest continuously conducted health survey in the world” (CDC 2014b).

## Trauma Informed Care (TIC)

Drawing on the ACE survey findings and those of many other childhood trauma studies, an orientation to service delivery has gained momentum that uses childhood trauma as a lens to understand the range of cognitive, emotional, physical, and behavioral symptoms seen when individuals enter systems of care. TIC comes from a values base of client safety and empowerment as well as an orientation to strong working alliances between clients and providers. DeCandia and Guarino (2015) have written a comprehensive review of the history and on-going development of the TIC orientation.

The Substance Abuse and Mental Health Services Administration (SAMHSA 2015) has defined four main points defining Trauma-Informed Care:

1. Realizing that trauma has a widespread impact on individuals, families, groups, organizations, and communities and has an understanding of paths to recovery;
2. Ability to recognize the signs and symptoms of trauma in clients, staff, and others in the system;
3. Integration of trauma knowledge into policies, programs, and practices;
4. Seeks to avoid re-traumatization

SAMHSA’s involvement in explicating TIC has raised awareness about the importance of a values-oriented approach to policies, practices and programs that help depathologize problemmatic behaviors. It offers strategies for creating service delivery climates of empathy and respect in work with individuals and families who have experienced traumatic events.

More recently, the contribution of neuroscience research has made its way into the social and behavioral health arenas by informing practitioners across disciplines about the impact of early trauma on the brain. Neuroimaging techniques such as magnetic resonance imaging (MRI) offer new understanding and validation of the impact of early traumatic events by focusing on brain development. Bridging the gap between academic studies and more popular publications, trauma-oriented, neuroscience-based information focuses on the neurobiology of distressing events and the subsequent detrimental impact they can have on social functioning (Child Welfare Gateway 2009). Early trauma has been found to cause changes in certain structures in the brain as well as alterations in chemical activity and these changes can result in heightened reactivity and impaired relational capacity (Phillips and Shonkoff, 2000).

Due, in part, to the varying attitudes about mental health disorders as well as mistrust of diagnostic labels Trauma Informed Care orientations have not been easily incorporated across cultures (Evans and Coccoma 2014). Studies assessing outcomes of TIC have also been lacking. A search of the literature for evaluation studies of TIC found one (Clark et al. 2008) that was a comparison of “consumer” attitudes toward social services that used a TIC orientation in the design of space and service delivery (called the “integrated condition”) and clients receiving care as usual. The analysis found that clients who received the “integrated condition” were more likely to report that services were trauma informed and that relationships with the service providers were more positive and characterized by respect for cultural identity. While the authors emphasize that the results are not predictive of treatment outcomes such as reduction of symptoms the positive experiences, particularly with the providers, can be a first step in healing.

There are risk assessment models currently in use in correctional facilities that do not explicitly focus on traumatic experience. The Risk, Need, Responsivity Model (RNR), for example, is an intervention that tabulates risk/needs factors as well as other attitudes and behaviors considered criminogenic (Andrews 2006). Unfortunately, the RNR model does not incorporate current neuroscience research that indicates the reactivity, impulsivity, and need for excitement that can result from early or even recent trauma. Instead, individuals with these symptoms are labeled in the RNR model as having an antisocial personality pattern (Bonta and Andrews 2007). Neuroscience research on the adolescent brain describes the drive for intense experience without regard for future consequences that so often characterizes adolescents to be the result, not of characterological or personality deficits, but of the mix of a combination of an increased number of dopamine receptors and surges in sex hormones (Steinberg 2014). Since so many incarcerated individuals are sentenced for crimes committed during adolescence it is important that neuroscience contributions become more widely incorporated in courtrooms and correctional facilities.

One probable reason for the lack of outcome studies that focus on symptom reduction in TIC-specific interventions is that TIC, as it currently exists, is primarily a set of information and values about working with individuals who have experienced trauma. This is an important framework that has promoted better working alliances and an empowerment focus but has not provided enough intervention-oriented guidance that would allow for outcome evaluation.

## Unintended consequences

The awareness brought by the ACE study and subsequent studies of early childhood trauma have been important and the benefits of incorporating Trauma-informed Care into services have shaped the environments in which services are delivered and heightened attention to the imperative to build client-provider relationships that build trust and a sense of empowerment. However, there are some serious issues that also arise with the attention they have brought by their focus on the impact of traumatic events. These issues are discussed below.

### 1. Over-attention to the negative

The ACE study and many, not all, of the studies that flow from it have a sole focus on the negative experiences of childhood. And the ACE survey of negative events was limited in the scope of types of adverse experiences it included. No data was collected in the ACE survey on protective or strength-oriented factors that may have been part of the lives of those in the sample. The consequence of attention to risks and problems to the omission of resilience and protective factors is a lopsided understanding of clients and this view becomes a limiting factor that can shape intakes, service delivery and research.

The powerful impact of the ACE study has generated other surveys that are also limited to risk factors. The CDC’s Behavioral Risk Factor Surveillance System (BRFSS) (2014a, b), for example, which is conducted throughout all 50 states in the U.S., the District of Colombia, as well as three territories, asks only one question that could be considered even slightly positive. Question 32b on the Women’s Version of BRFSS asks, “How many close friends or relatives would help you with your emotional problems or feelings, if needed?” This could be considered positive because it asks about close friends or relatives. Unfortunately, the question is oriented toward having a problem. A different version of the question or a separate question could be, “How many close friends or relatives do you have supportive relationships with?”

No matter how vulnerable a person or family is they also have strengths, they have dreams for the future, they have bounced back from challenges. It is not that the exclusion of strength-based or resilience information is an intentional omission in so many programs. It is that the Trauma Orientation seems to create a single-point focus that overrides or edges out an inclusion of and attention to strength-based information in many research studies and other information-gathering programs.

A factor that contributes to this Trauma Orientation in intakes is that most social service workers are in organizations that are under-resourced in terms of time and staff. This time/staff squeeze contributes to an urgency to get “to the heart of the matter,” which is the problematic events that have happened or are still happening to a client. And, clients are expecting that focus. But, the true “heart of the matter” is the resilience that a person retains in the face of many challenges. Those factors that contribute to resilience are the factors it is important to know about. They have shaped resilience and can help amplify it when enlisted during service delivery.

Inclusion of strength-based questions is important in many ways: 1) It allows the person responding to the form or interview to feel known in more ways that just the negative events of life and the corresponding problems; 2) it gives a fuller picture to staff so that the likelihood of “armoring,” the hard shell that workers can develop when faced with client problems that seem insurmountable, is diminished and a sense of manageability increases; 3) it increases the likelihood that the strengths can be used during the service delivery process; 4) in research it provides richer understanding of the relationship between the independent and dependent research variables and can increase the explanatory power of the analysis. For example, in the ACE study not all individuals with higher ACE scores experienced the many health risks, some didn’t. It would be helpful to know if the reason for the difference is the protective factors in their early lives. How many protective factors, or which ones, diminish the effect of adverse experiences? Those factors, unfortunately, were not collected.

The type of protective questions that could enrich the ACE survey includes, “In your childhood was there a person or persons in your family who took a positive interest in you?” Or “Did some people in your family look out for and support each other sometimes?” Or “Were there some things as a family you enjoyed doing together?” They would include questions beyond the family since they, too, can contribute to resilience: “In your childhood was there a person or persons outside the family who supported you? Motivated you? Seemed to appreciate your strengths?” Questions such as these can be interspersed with questions about adversity (Leitch 2015).

Fortunately, more recent risk assessment instruments move beyond a Trauma Orientation to include positive or protective factors (Rains and McLinn 2013). Thompson (2010), in a doctoral thesis, discusses the history of and theoretical models guiding resilience-oriented surveys, including definitional issues and domains. And, some surveys have moved beyond an exclusive focus on family experiences to include a much richer focus that captures school and community experiences as well. For example, The Annie E. Casey Foundation’s Evidence2Success Youth Experience Survey (2013) outlines key risk and protective factors specifically developed for assessment and intervention by communities. The survey includes risk questions such as, “How wrong do your parents feel it would be for you to smoke marijuana?” and, “How many times have you changed homes since kindergarten?” and, “In the past year (12 months), how often have you been treated badly because of your race?”

Examples of protective questions include: “Do you share your thoughts and feelings with your mother (or the person who is like a mother to you)?” and, “How often do your parents (or caregivers) tell you they’re proud of you for something you’ve done?” and, “In the past year how many of your best friends have participated in clubs, organizations, or activities at school?” The Evidence2Success is an example of a survey that focuses on the ecology of a child’s life; including questions and statements about school and community relationships in addition to a family focus.

Collecting resilience information in addition to adverse experiences can increase the richness of studies measuring the impact of program interventions. It can guide analyses that examine the mediating effect of protective factors on adverse events. It can refine analyses by examining whether there are “windows of opportunity” when protective factors have a larger impact or whether there differential effects of some protective factors (e.g., family factors, community factors, peer factors). And, when attrition from a study or program is reduced because participants feel better understood there will be a more reliable understanding of what should be replicated in program design and a far better knowledge base about the characteristics of clients that appear associated with better or worse outcomes.

### 2. Ethical issues

In collecting data from anyone, but particularly from individuals who are vulnerable, it is essential to pay attention to the potential for re-traumatization during information gathering. The method of data collection and the content of the items are dimensions of human subjects protection that must be considered.

The ACE survey and the BRFSS, are both large surveys that collect only trauma-specific data, and are not administered face-to-face. The ACE survey was collected by mail and the BRFSS as a telephone survey. What is the effect on research participants when only questions about risk factors (spanking, suicidal thoughts, sexual abuse, etc.) are the focus? If a respondent is upset after receiving the ACE form by mail or the BRFSS call who would know? Is there a follow-up call if the information triggers intense feelings and memories? Is there a procedure for checking back with respondents to find out?

SAMHSA guidelines emphasize avoiding the re-traumatization of clients (SAMHSA 2015). Institutional Review Boards (IRB) must consider the potential for re-traumatization when only emotionally charged questions are used in a mail or telephone survey. No information on follow-up with respondents could be located on this important human protections issue. Research is needed that examines the impact of trauma-oriented surveys on respondents, including on sample attrition.

### 3. Relationship and manageability

It can be a challenge to attract and maintain vulnerable individuals in services who are not court-ordered. Clients might present once and not return or, after a telephone intake, they may not come in for an initial session. Over-attention to negative symptoms and the exclusion of positive qualities and protective events that characterizes so many intake processes may be a contributing factor.

For example, if a teen client has been a run-away since age 10, trafficked for sex since she was 12 years old, raped numerous times, bears the tattoos of “pimp ownership” on the back of her neck, and is alternately hostile and withdrawn a worker can feel anxious, overwhelmed, and even adversarial. As mentioned earlier, neurobiological studies of childhood trauma highlight the relational difficulties of many trauma survivors. And these relational challenges can be seen in the ways a trafficked teen presents during intake and early services.

When the intake form for an agency working to engage sex-trafficked teens in services was changed to include questions about positives, workers began to feel a sense of hope and believed the teens were more likely to return for a second meeting (Leitch and Snow 2010). An example of a question asked in the revised intake to build more complete knowledge of the teen was, “If your good friend was here with us today and I asked her what she likes best about you, what would she say?” Questions like this one can change the quality of the exchange, decrease suspicion and hostility, and remind both the client and the worker that she is more than a sex-trafficked person with multiple arrests.

Bessel van der Kolk (2014) highlights the need to look at the *ecology* of lives as a richer way to understand individuals, moving beyond an over- focus on the negative. When the emotionally challenging details of the traumatic experiences, which are required in order to convict a sex-trafficking perpetrator, can be balanced with strength-oriented questions about the teen trust and safety can slowly be built and worker overwhelm and defensive amoring decreases. These, in turn, can enhance the stability of both the teen and provider.

### 4. Generating and reinforcing dysregulation

Another compelling reason for intentionally weaving strengths into both research and practice comes from what neuroscience research has found about the human nervous system and its powerful role in the regulation of physical, emotional, and cognitive functioning (Cozolino 2002; van der Kolk 2014).

Information gathering processes can be developed in a way that mimic the rhythm of the Autonomic Nervous System when it is in a healthy, regulated balance. This means creating a rhythm of calming and activating questions throughout the process: ask a few questions that generate Parasympathetic calming followed by a couple of questions that might be activating, followed by a calming question, and so on. Workers can learn to track the patterns of activation and calming by noticing such sensory details as breathing, muscle tension or relaxation, facial coloration, posture and gestures. This sensory information can help guide the decision to shift from activating to calming questions and decrease the potential for dysregulation.

### 5. Information is not enough

Sensitivity to the impact of traumatic events that flows from TIC, while helping to reduce pathologizing and enhance relationship, does not always help workers know what to *do* beyond that. Another way to describe this is that TIC provides information on the kinds of experiences that result in dysregulation and the corresponding array of symptoms but usually does not provide enough specificity about how the mind-body system is designed to respond to threat and fear (as well as the potential for resilience). This limits service providers’ ability to design trauma and resilience-informed interventions that link the mind and the body that can decrease reactivity. Particularly lacking are interventions that use practical skills to promote the capacity for self-regulation. An essential building block of wellbeing as well as mastery and dignity is knowing how to modulate your own reactivity. This is true for providers as well as clients.

### 6. Lack of neuroscience-based information in understanding trauma

Too few interventions that are designed for rehabilitating offenders, who are often susceptible to poor impulse control and the corresponding attitudes and behaviors, have incorporated recent research from neuroscience about the impact of trauma on the mind-body system. When these symptoms and behaviors are viewed from a neurobiological lens that highlights how the human nervous system is wired to respond to threat and fear the use of negative labels decreases and the focus is on finding ways to bring the nervous system back into balance.

The incorporation of neurobiological knowledge facilitates the design of skills-based interventions suitable across cultures and with groups that may stigmatize or not have access to or willingness for counseling, since all humans, regardless of culture, gender, race/ethnicity, are wired similarly in their response to perceived threat and fear. These interventions target regulation of the nervous system rather than putting a primacy on insight and emotion. (Levine 1997; Ogden et al. 2006; Leitch et al. 2009; van der Kolk 2014). Cognitions and emotions are included in these approaches but are secondary.

Further evolution of TIC can be greatly enriched by the incorporation of findings from neuroscience research that currently are absent in most approaches. Expanding the TIC knowledge base enables the design of a wider more culturally-sensitive range of intervention, including teaching self-regulation skills for use in self-care as well as peer-to-peer. Three of these key concepts from neuroscience that could enhance TIC are described below.

#### a. Autonomic Nervous System (ANS)

Information about the Autonomic Nervous is a core element in understanding of how the mind-body system responds to threat and fear as well as how to amplify resilience. Like many aspects of nature that have rhythms and cycles, the human body also has them. One rhythm in particular that is accessible to intervention and extremely potent in its influence on health and wellbeing is the rhythm between the two branches of the Autonomic Nervous System (ANS), the Sympathetic and Parasympathetic branches.

**Fig. 1:**
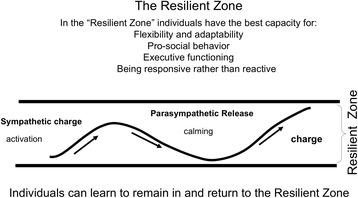
ANS Rhythm in the Resilient Zoneᅟ

With the advent of increasingly sophisticated fMRI techniques and interpretation of results the past decade there has seen increasing information about the ANS and its two branches. They work in a rhythm with each other; most simply put, the Sympathetic is the activator and the Parasympathetic is the calmer. When the two are in an optimal rhythm or balance the individual can be responsive to life events rather than reactive to them. When the ANS is in a healthy balance, which can be called the Resilient Zone, there is access to a conscious system of information processing in which stress chemicals do not block access to the cortex, or thinking part of the brain. This promotes better capacity for problem-solving and strategic thinking in stressful situations rather than reactivity (Roozendael, McEwen, & Chartarji (2009) as well as the ability to engage in pro-social behaviors. Figure 1 below illustrates ANS rhythm when it is in the balance:

The Autonomic Nervous System (ANS) influences all of the organs in the body (Schmidt and Thews 1989). That is one reason why distressing events, such as those from the ACE study, are associated with mental and physical health problems. When stressful, distressing, and traumatic events bounce an individual out of the Resilient Zone the dysregulation that occurs can lead to physical, emotional, cognitive, and behavioral symptoms that affect health and well-being in many negative ways (Scaer 2005; van der Kolk 2014).

**Fig. 2:**
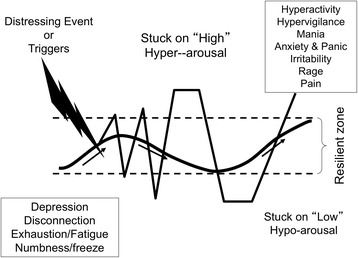
ANS Rhythm Outside the Resilient Zoneᅟ

And, when individuals experience a repetitive or cumulative series of negative experiences it can “wire in” the dysregulated rhythm (Scaer 2005), leaving them reactive and stuck in a state of either hyperarousal (being bumped above the Resilient Zone) or hypoarousal (stuck below the Resilient Zone) or oscillation between the two extremes as the nervous system attempts to find balance Fig. 2.

The graphic above shows the disrupted rhythm of the ANS and examples of what can happen when someone is stuck on “high” or “low.” In addition to the symptoms in the chart that can result from being outside the Resilient Zone, stress has an impact on memory. Neurochemicals such as adrenalin, which are generated in response to perceived threat, help to etch a distressing or traumatic event into memory. However, “high arousal disconnects brain areas necessary for proper storage and integration of information” (van der Kolk 2014:176). The result can be fragmented and distorted memory.

Intake processes, courtroom testimony, evocative and intense interventions such as Prolonged Exposure Therapy, and research questionnaires that focus only on adverse experiences and symptoms have the potential to bump people into states of reactivity. Neuroscience research has shown that when individuals are in these states of dysregulation memory, concentration, and attention are negatively affected (Lutz et al. 2008).

The implications of this information should be considered in courtrooms where the legal process is designed to be adversarial. It can help understand why a rape survivor, when under cross-examination, may change details in the story of what happened, have trouble identifying the accused, and describing other specifics of a crime that shape jury decisions.

Behavior is also shaped by physiological reactivity. There tends to be a decrease in pro-social behaviors such as collaboration and kindness in individuals when bounced outside the Resilient Zone since those usually require full cognitive capacity and the corresponding ability to *respond* rather than *react* to life events. When ANS rhythm is outside the Resilient Zone there can be increases in such behaviors as substance abuse, self-harming, family violence, poor school and work performance, bullying, and social disengagement, to mention only a few.

Graphics like the two above help clients and caregivers understand the reason for their responses to stress, distress, and trauma. The information, which can be referred to as neuroeducation because of its focus on education about the neurobiology of threat, fear, and resilience, can be useful in motivating individuals to pay attention to the body’s signals of distress and calming and to motivate practice of self-regulation skills. It becomes a way for those who have been cut-off from the body’s signals of distress to pay attention at this essential sensory, “bottom-up” level and use skills to return to balance.

The graphics can be shared with individuals (clients and staff) and used in creating resilience-oriented policies, programs, and, most importantly, actions. The information in the graphics can be used in work with clients, work teams, and communities to provide a rationale for the use of self-regulation skills.

Neuroeducation helps individuals understand what was happening in the nervous system when they reacted to a threat in a way that got them into trouble. It focuses on biology rather than pathology. The neuroeducation can also motivate individuals to learn and practice skills-based approaches to self-regulate so their reactivity diminishes and they have a deeper “Resilient Zone” due to neuroplasticity. Inside the Resilient Zone there is greater potential for pro-social behaviors such as collaboration, empathic responses, future-oriented planning, etc.

#### b. The fast and slow systems of information processing

A second important concept from the neuroscience labs that can amplify the contribution of TIC is that every individual is wired with defensive responses (Fight, Flight, Freeze, and Tend and Befriend) that can be automatically and unconsciously triggered by even the *perception* of threat. This appraisal of threat takes place initially below the level of consciousness and is entirely subjective. What one person would perceive as threatening another might perceive as an exciting challenge. And, because individuals are quite elegantly wired to maximize survival, the brain has two processing speeds that function to make sense of in-coming information and to take action in behalf of survival: the “fast system” and the “slow system” (Kahneman 2013).

In the fast system, problem-solving processes are blocked by neurochemicals in order to save valuable seconds and increase survival chances. If, for example, a speeding car jumps the curb and comes at someone on the sidewalk the time to think about escape options may result in injury or death. Instead, the individual is automatically launched into flight mode, instantly leaping out of the way. This is the fast system in action, protecting survival.

However, fast system processing can cause problems when an individual is triggered by an event from the past. For example, if a correction officer is triggered (due to his own previous trauma) by the sound of a scuffle behind him and instinctively goes into a defensive response of fight by hitting a handcuffed prisoner, that fast system of processing can result in career-risking behavior. And, if nearby officers go into fast system processing and shut down, they can be deemed “psychologically” unprepared and also be subject to disciplinary action.

In the slow system the threat is unconsciously appraised as manageable and cortical thinking is not blocked by neurochemicals. Conscious problem-solving and decision-making can then occur from inside the Resilient Zone. Perceived threat generates fast system processing and *reactivity* (Kahneman 2013). In some cases the fast system of processing protects survival but in other situations it leads to risky or shame inducing behaviors.

Neuroeducation about the fast and slow systems of processing can help reduce acute distress in, for example, a Correction Officer who is filled with shame because he froze during a lethal situation and his buddy was badly wounded as a result. It can help understand why a police officer may have shot 16 times at a youth who was running away. The information does not release individuals from the need to take responsibility for the impact or outcome of these fast-system actions; but it can help understand the neurobiological dynamic behind the action and reduce the labeling of the behavior as characterological.

And, when neuroeducation is channeled into action it can change organizational practices. For example, by using neuroeducation, the process leading up to invasive body-checks with incarcerated people can be redesigned in a way that decreases the likelihood of the prisoner going into fast system reactivity that gets him or her into additional trouble.

#### c. Neuroplasticity and self-regulation

Another neuroscience finding that can contribute to a shift from information to action in TIC is the ability of the brain to change. In the past, the belief was that the brain was fully developed by early adulthood. It is now broadly recognized that the brain is able to change over the lifespan, for better or for worse (Doidge 2007). And, what neuroscience has shown makes the difference in whether the plasticity change is beneficial or not is how and to what we pay attention.

Neuroplasticity, can be enlisted in building pro-social behavior as well as emotional and physical well-being by skills that teach self-directed attention. New neurons are generated (neurogenesis) and reinforced (neuroplasticity) during learning and practice; and a key element of learning comes from attention (Citri and Malenka 2008). Self-directed attention practices, including but not limited to various forms of meditation, have been found in hundreds of studies to promote improved health, compassion and collaboration, and a range of other well-being indicators (Grossman et al. 2004; Jacobs et al. 2011).

A key driver of neural connectivity that can enhance neuroplasticity in ways that deepen resilience has been found to be the monitoring and training of attentional focus (Lutz et al. 2008; Tang et al. 2007; Tang et al. 2014). The majority of studies showing ways to enhance connectivity using attention-based networks comes from meditation studies. Understanding the neural mechanisms underlying attentional practices has been steadily growing. However, a limitation in the research is the lack of studies that discriminate between different forms of attention-based practice (Chiesa 2012) whether in a meditation-based model or in attention training, like the self-regulation skills training proposed in this paper, that don’t require meditation. Like meditation, self-regulation skills train attention and teach people how to redirect and sustain attention in particular ways that can be used prior to and during challenging events as well as practiced over time to build deeper nervous system balance via neuroplasticity.

Self-regulation skills do not focus on insight or clinical interpretation (Levine 1997; Ogden et al. 2006; Leitch et al. 2009). The skills rely on the individual directing attention to patterns of activation and calming in the body. The focus is on the rhythm of the Autonomic Nervous System as reflected in such sensory experiences as, for example, quality of breath, heart rate, and muscle tension and relaxation patterns. When activation goes outside the Resilient Zone particular skills are used to return to the Resilient Zone and to reinforce the experience of balance.

The two primary objectives of self-regulation skills are 1) to have a practical, immediate way to manage and reduce states of distress and activation that can be used independently as well as in clinical intervention and 2) to use neuroeducation to help understand symptoms and behaviors and to motivate practice of the skills in order to utilize neuroplasticity to wire-in greater resilience and decrease the power of stressors to trigger reactivity. The skills explicitly incorporate strengths and protective factors in the process of self-regulation and generate a sense of mastery and efficacy. They can be used for provider self-care as well as in work with clients. They can also be used peer-to-peer.

Neuroplasticity is a hidden asset in human potential. It can be accessed by an individual’s conscious or unconscious patterns of attention. An old adage says, “Where your attention goes, energy flows, and that’s what grows.” Knowledge about neuroplasticity has been an underutilized mechanism of positive change in most social services approaches, both for provider self-care and in work with others. Developing self-regulation skills that can be practiced independently by providers and clients alike can decrease reactivity and increase slow system processing in addition to building a sense of mastery and self-control.

### Using ACE findings and neuroscience: moving from information to action

The neuroscience concepts above help shed light on how cumulative adverse childhood experiences can maintain the brain in a threat-oriented mode which, over time (through neuroplasticity), can wire in a level of physiological reactivity; a reactivity that can last throughout adulthood, creating physical and emotional health problems and repeatedly cause problematic behaviors. A vicious cycle is put in place and reinforced. This reinforcement process has been described as the body re-setting itself in a way that the world is experienced as a dangerous place (van der Kolk 2014).

The prison system is an example of the ways undigested trauma from early childhood experiences can join with the conditions of harshness and violence in many of our U.S. prisons and contribute to reinforcing a cycle of reactivity in both Correction Officers and prisoners. The correctional system is rife with challenges to the health and well being of Correction Officers (COs) as well as prisoners. Suicide rates of COs are more than double that of police officers as well as for the national average (Steele 2009) and their average life expectancy is 59 years old (Cheek and Miller 1982; Steele 2009). How much is due to adverse childhood experiences? How much is due to our system of incarceration, which can create a culture of violence in which both the imprisoned and those in charge of them must operate in a perpetual state of hypervigilence and wired-in reactivity? Practices throughout the criminal justice process can benefit from information from neuroscience as well as the skills that are based on this information to create environments and approaches that enrich rather than deplete the ability of both COs and inmates to self-regulate as a core practice. Practical self-regulation skills that are based on neuroscience research belong in police and CO training academies, and with other first responder groups as a tool to build resilience and decrease reactivity during stressful situations.

## Conclusion

The ACE Study and Trauma-Informed Care have made a strong and positive contribution to understanding the powerful role and negative health effects of adverse events in childhood. The effects of early negative childhood experiences are found to carry on throughout adulthood, even affecting life expectancy. The two contributions have helped sensitize service providers to the risk factors that shape behaviors and health, have helped policy makers and service providers shift away from a characterological lens of human behavior to one that recognizes the impact of early and traumatic experiences, and have highlighted the importance of early childhood prevention programs and family support.

The unintended consequences, however, have contributed to an over-focus on negative events to the neglect of protective and positive factors. This over-focus, while not characterizing all policies and programs, is still too common, nevertheless. It has shaped research as well as social programs. During service delivery, collection of the adverse details about people’s lives is often *necessary but it is not sufficient*. A focus on individuals’ strengths and competencies is essential. And, Trauma-Informed Care is also *necessary but not sufficient.* Policy makers and providers must know what to do with the information, what actions are needed. Action-oriented interventions will facilitate evaluation studies of outcomes. This will advance the field of TIC.

Current neuroscience-based information (“neuroeducation”) has an important role to play in the field of criminal justice including 1) redesigning information gathering processes to decrease re-traumatization, 2) decreasing the use of labels such as “anti-social” that do not take into account the neurobiological effects of trauma on the nervous system, 3) the incorporation of self-regulation skills training for providers and clients, and 4) facilitating outcome evaluations of trauma and resilience oriented skills-based programs. Drawing on neuroeducation about nervous system activation and calming as well as slow and fast systems of information processing can decrease the potential of both data collection and social programs to re-traumatize clients and research subjects and can help reinforce nervous system stabilization.

Practical skills, based upon key concepts from neuroscience can, as a next step, move Trauma-Informed Care beyond *information* to *action* by building the capacity for self-regulation. Greater attention to strengths and protective factors as well as challenges can reorient the way that researchers and practitioners collect information, design interventions, conduct data analyses, and support the dignity and trust of clients.

Using non-clinical, skills-based approaches individuals (clients as well as service providers) can learn to assess the state of their nervous systems and direct their attention using practical skills that promote self-regulation and deepen resilience. And, researchers can adapt the idea of reinforcing a Resilient Zone nervous system rhythm when designing the patterns of questions in surveys and interviews.

The increased attention to traumatic experiences from the ACE study and the expansion of attention in more recent surveys to collect protective factors as well as risk factors has offered an essential understanding about the power of experience to affect health, behavior, and well being. When that knowledge is combined with neuroscience-based skills, trauma informed approaches will move from information to measurable action.
